# Novel deep learning method for coronary artery tortuosity detection through coronary angiography

**DOI:** 10.1038/s41598-023-37868-6

**Published:** 2023-07-10

**Authors:** Miriam Cobo, Francisco Pérez-Rojas, Constanza Gutiérrez-Rodríguez, Ignacio Heredia, Patricio Maragaño-Lizama, Francisca Yung-Manriquez, Lara Lloret Iglesias, José A. Vega

**Affiliations:** 1 Advanced Computing and e-Science Research Group, Institute of Physics of Cantabria (IFCA), CSIC - UC, 39005 Santander, Cantabria Spain; 2 Facultad de Medicina, Universidad Católica del Maule, Talca, Chile; 3 Departamento de Morfología y Biología Celular, Grupo de Investigación SINPOS, Universidad de Oviedo, 33006 Oviedo, Principality of Asturias Spain; 4 Facultad de Ciencias de la Salud, Universidad Autónoma de Chile, Talca, Chile; 5 Department of Hemodynamics, Talca Regional Hospital, Talca, Chile

**Keywords:** Anatomy, Cardiology, Interventional cardiology, Computer science, Statistics

## Abstract

Coronary artery tortuosity is usually an undetected condition in patients undergoing coronary angiography. This condition requires a longer examination by the specialist to be detected. Yet, detailed knowledge of the morphology of coronary arteries is essential for planning any interventional treatment, such as stenting. We aimed to analyze coronary artery tortuosity in coronary angiography with artificial intelligence techniques to develop an algorithm capable of automatically detecting this condition in patients. This work uses deep learning techniques, in particular, convolutional neural networks, to classify patients into tortuous or non-tortuous based on their coronary angiography. The developed model was trained both on left (Spider) and right (45°*/*0°) coronary angiographies following a fivefold cross-validation procedure. A total of 658 coronary angiographies were included. Experimental results demonstrated satisfactory performance of our image-based tortuosity detection system, with a test accuracy of (87 ± 6)%. The deep learning model had a mean area under the curve of 0.96 ± 0*.*03 over the test sets. The sensitivity, specificity, positive predictive values, and negative predictive values of the model for detecting coronary artery tortuosity were (87 ± 10)%, (88 ± 10)%, (89 ± 8)%, and (88 ± 9)%, respectively. Deep learning convolutional neural networks were found to have comparable sensitivity and specificity with independent experts’ radiological visual examination for detecting coronary artery tortuosity for a conservative threshold of 0.5. These findings have promising applications in the field of cardiology and medical imaging.

## Introduction

Worldwide, coronary artery disease (CAD) resulting in heart failure is one of the most frequent causes of premature death^1^ accounting for 30% of deaths in 2014 in the United States^2^ and 45% of deaths in Europe, with an estimated cost of €210 billion per year for the European Union alone^3^. Thus, detailed knowledge of coronary artery morphology is essential for planning any interventional treatment of CAD, such as stenting, stent sizing, or decisions regarding these procedures.

To assess coronary artery anatomy, both in normal and pathological conditions, coronary angiography (CAG) is considered the gold standard, despite some potential risks such as ionizing radiation, invasiveness, and a small associated risk of morbidity^4,5^. Nevertheless, visual evaluation of CAG conditions may vary between observers. In this regard, a recent study by^6^ analyzed both interobserver variability and consistency between operator estimation and quantitative measurements of CAG analysis. They concluded that visual assessment of CAG may overestimate a CAG lesion and thus lead to unnecessary interventions. To solve these troubles and accurately evaluate normal and pathological morphology of CAG, automated measurement systems can be introduced. Hence, in this context, Artificial Intelligence techniques, in particular deep learning (DL) methods, can play a key role in CAG analysis.

One of the most important parameters to assess in CAG is the so-called arterial tortuosity (AT; CAT: coronary artery tortuosity) which may be a marker of vascular fragility or a useful indicator of underlying arteriopathy. AT can be defined as an exaggerated S- or C-shaped curvature, an acute angulation, or a circular loop in the course of an artery^7^. CAT is considered when there are at least three marked curves in any section of a coronary artery during both systole and diastole. Each of these curves shows a change in direction of at least 45 degrees compared to the normal direction of the coronary vessel^8^. CAT is a common finding in CAG with a prevalence of 15–40%^9^ that is rarely reported by cardiologists^10^. However, CAT is associated with reversible myocardial perfusion defects and with stable angina, angor pectoris and spontaneous coronary artery dissection^11,12^. Therefore, accurate detection of CAT in CAG to prevent these cardiac lesions is of utmost interest.

In this paper, we report the development of a DL system through convolutional neural networks (CNNs) to detect CAT. The performance of the DL system is further compared with independent experts’ radiological visual examination (RVE). To our knowledge, this is the first study on the application of DL techniques for CAT detection in CAG, which could offer promising applications in cardiology.

## Methods

We propose a classification convolutional neural network to perform CAT detection from CAG. The code is based on the image classification module available in the DEEP Open Catalog^13^. The original classification model developed in the DEEP framework was adapted to our specific task.

### Data acquisition

This is a retrospective study approved by the Human Research Ethics Committee of the Maule Health Service and the Ethical Committee for Biomedical Research of Talca Regional Hospital, Chile. All methods reported in this work were carried out in accordance with the pertinent guidelines and regulations. Since this study was approved by the Ethical Committee for Biomedical Research of Talca Regional Hospital, without direct interaction with patients, informed consent was not required.

#### Population

This is a retrospective clinical study in a sample of 18,000 patients who were referred between 2016 and 2022 to the hemodynamic unit of the regional hospital of Talca with symptoms of coronary disease. The subjects underwent CAG with a diagnosis that did not reveal significant angiographic lesions, i. e., patients did not report a coronary alteration associated with the clinical condition for which the examination was indicated. The patient population came from Chile, with a mean age of 68 years (SD 8 years), and comprised 216 men with a mean age of 69 years (SD 9 years) and 185 women with a mean age of 68 years (SD 6 years). Among these patients, 658 CAG were considered according to the inclusion and exclusion criteria, as explained in Section “Initial inclusion and exclusion criteria”. CAG of the participating patients was obtained anonymously. Medical records were retrieved from the database of the Regional Hospital of Talca. Comorbidity was not considered in patient selection.

#### Initial inclusion and exclusion criteria

The initial inclusion criteria were as follows: subjects without disfiguring angiographic lesions or significant anatomical variations, left 45°*/*25° (Spider) and right 45°*/*0° projection, in which angulation dispersion was not greater than 3°. Exclusion criteria were subjects with left ventricular hypertrophy, valvular heart disease, anatomical variations of the coronary arteries, deforming coronary anomalies, a history of previous CAG, cardiomyopathy or history of other heart disease, as these patients may have pathologically abnormal coronary arteries. Most of these criteria were proposed by^14^. After applying all the inclusion and exclusion criteria, the final sample consisted of 658 CAG images.

#### Calibration, patient and image selection

The collection of images corresponding to the selected patients was obtained from the database of two different angiographers belonging to the hemodynamic unit of the Regional Hospital of Talca. Images from 2016 to 2019 were obtained from a Siemens^®^ angiographer (95 CAG), while images from 2019 to 2022 came from a Phillips^®^ angiographer (563 CAG). Only the images corresponding to 45° left/25° caudal projection (Spider) for the left coronary artery, and 45° left*/*0° the for right coronary artery projection were selected.

A single image capture was obtained from each angiographic film. The file was saved in jpg or png format for the left and right CAG at the point of maximum arterial contrast filling. Then, if necessary, the image was subjected to artifact removal, since an external object would interfere with the interpretation of our neural network model. Coronary artery tortuosity (CAT), which was identified by the presence of three or more consecutive kinks (defined as a 45° change in vessel direction) along the main trunk of at least one major epicardial coronary artery, was considered to label an image as corresponding to a patient with CAT.

Fifty angiographic images from the total set that met the pre-established inclusion and exclusion criteria were used for calibration. As reference values, the results obtained by three experts in the field (three cardiologist-angiologists) were considered, reviewed by a cardiologist, who used the following qualitative methods: (a) visual examination of the vessel’s tortuosity by defining the fixed anatomical points within which tortuosity is measured, (b) recording of the number of inflection points between fixed anatomical points, (c) counting of the number of kinks and loops, and classification according to defined tortuosity criteria and, (d) associating tortuosity with arterial elongation and wall weakening observed as a minor change in wall contrast uptake. This initial calibration set had a reliability of the biometric analysis of 98*.*0% for inter- and intra-examiner values using intraclass correlations (ICC).

#### Ground truth

The three cardiologist-angiologists, who had been trained with the fifty angiographic images used for calibration, served as ground truth (ideal expected outcome used to calculate the accuracy of the DL algorithm) and evaluated CAG during the study period to find CAT among patients. The ground truth was established independently by one of the three cardiologist-angiologists, who had an average of 15 years (12, 16 and 17 years respectively) of experience, and a mean of seven thousand tests performed.

### Dataset

Our final experimental dataset consisted of 658 CAG images, corresponding to 401 different patients in total. Table 1 depicts the number of patients available for each type of CAG.

**Table 1:** Total number of patients available for each coronary angiography.

Side	# patients with coronary artery tortuosity	# patients without coronary artery tortuosity
Left or spider	182	217
Right or 45°/0°	52	207

### Model design

Convolutional neural networks (CNNs) are a type of DL neural networks specifically designed to analyze images, both with numerical (regression) or categorical (classification) labels. In the present study, we had to solve a binary classification problem on CAG images, detecting either patients with coronary artery tortuosity (CAT) or patients without coronary artery tortuosity (WCAT).

There are three sets into which the data (in our case, images) are subdivided to be used in a DL model: training, validation, and test. The training set is utilized to train the model. During the training phase, the hyperparameters are tuned in order to optimize the model’s performance over the validation set. In case the accuracy over the validation set stops increasing at a predetermined number of epochs in the training phase, the training is stopped. This is one of the most common regularization techniques in DL, which is known as early stopping^15^. The test set contains images that the model has not seen before and, thus, it is used to assess the final unbiased accuracy of the model.

The procedure to detect CAT first consisted in training a CNN model with CAG images. We trained five different models following a fivefold cross-validation strategy^16^. The total number of patients was balanced in each of the fivefold sets (training, validation, and test), which means that half of the images corresponded to patients with coronary artery tortuosity (CAT) and the other half to patients without coronary artery tortuosity (WCAT) (either Spider or 45). The images were randomly selected from any of the angiographers, and there were 450 images for training (225 CAT, 225 WCAT), 46 for validation (23 CAT, 23 WCAT) and 48 for testing (24 CAT and 24 WCAT), keeping the same distribution in each of the cross-validation folds. As the number of WCAT images was higher than the number of CAT images, we randomly repeated 38 of the corresponding CAT images in training, in order to use all the WCAT images available. Both coronary artery projections were included in the same CNN, as we performed several tests to evaluate whether the DL model performed better when training each projection separately or with both, and found that it showed similar validation metrics. We decided to train with both left 45°*/*25° (Spider) and right 45°*/*0° projections to include a larger number of images during training, thus, the model could more precisely learn the differences between CAT and WCAT.

We used an Xception^17^ CNN with images of size 528 × 528 pixels. In our method, CAG images were resized to meet this requirement. After trying different model initializations, the batch size was finally set to 16, the number of epochs was fixed up to a maximum of 50, although we used early stopping to prevent overfitting (setting patience in 15 epochs), with the result that the number of training epochs was lower in our models. We also employed Adam optimizer^18,19^ to speed up the training. Initially, a pretrained ImageNet base model was loaded to optimize the learning task. This methodology is known as transfer learning^20^. We took the ImageNet pretrained model and substituted the last Fully Connected (FC) layer with a FC layer adapted to our problem (binary classification). Then, we trained everything end-to-end, but the base feature extractor was trained with a much lower learning rate compared to the FC layer, which started from random weights. Fine-tuning the feature extractor makes it more relevant to the features present in this specific problem that might not appear in ImageNet^21^.

**Figure 1:**
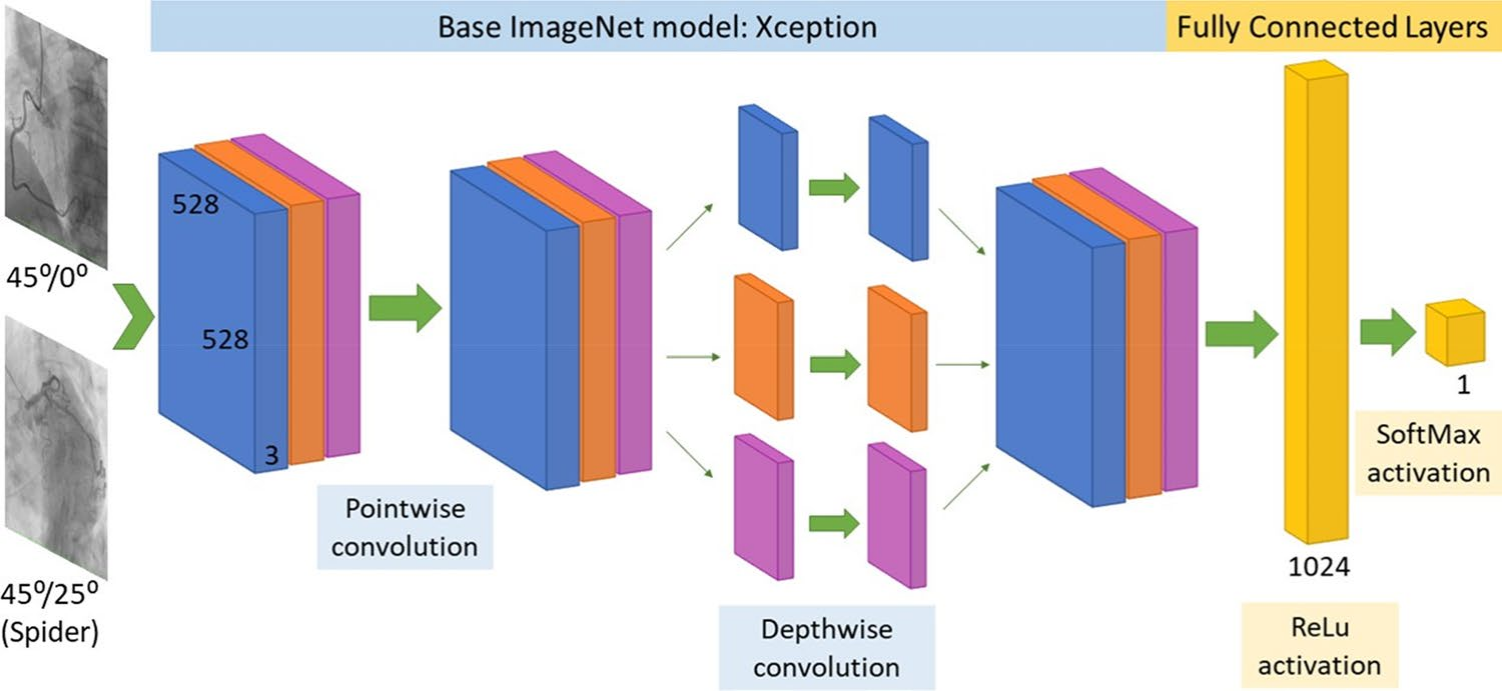
Proposed CNN architecture for coronary artery tortuosity detection.

The deep learning architecture developed in this study to detect CAT is illustrated in Fig. 1. As the total number of available images was quite limited, we used data augmentation^22^ in both training and validation sets to improve our models (see Table A1 for further information).

Each model was trained using a Tesla V100-PCIE-32 GB GPU. The model was coded using Keras^23^ and TensorFlow version 1.14.0^24^ in Ubuntu 18.04.2 LTS.

### Statistical analysis

The proposed fivefold cross-validation model to detect CAT was evaluated using the following statistical classification measures: accuracy, sensitivity, specificity, positive predictive values (PPV), negative predictive values (NPV), F_1_ score and area under the receiver operating characteristic curve (AUC). The AUC was calculated considering the corresponding probabilities of each predicted label. The accuracy, sensitivity, specificity, PPV, NPV and F_1_ score were calculated considering the label most likely to be predicted by the model. The operating threshold for deeming a label as a prediction of the model was established at 0.5.

### Ethical approval

This study was approved by the Human Research Ethics Committee of the Maule Health Service and the Ethical Committee for Biomedical Research of Talca Regional Hospital, Chile.

## Results

Different experiments were conducted to assess the quality of our method. The independent 5-Fold CNN models were trained and evaluated on the corresponding test set, performing a statistical analysis and a saliency maps examination to visually verify the predictions of the DL model.

### Deep learning system performance evaluation

To evaluate the performance of the DL model, we calculated the mean and standard deviation (SD) of the statistical measures described in Section “Statistical analysis”. Table 2 shows the results obtained after this calculation.

**Table 2:** Classification metrics for detecting coronary artery tortuosity in coronary angiography with the proposed deep learning system.

Metric	Mean	Standard deviation
Accuracy	0.87	0.06
Sensitivity	0.87	0.10
Specificity	0.88	0.10
PPV	0.89	0.08
NPV	0.88	0.09
F_1_	0.87	0.07
AUC	0.96	0.03

Furthermore, we repeated the predictions of our model on a test subset considering different contrast factors^25^ in CAG preprocessing (in particular, 0.5, 0.75, 1.0, 1.25, 1.50, 1.75 and 2.0), in order to assess whether adjusting the contrast had an impact on the predictions. However, the statistical metrics did not show significant variations. Therefore, we suggest that additional CAG preprocessing before feeding our deep neural network model does not influence the outcome of the predictions.

### Comparison with independent radiological visual examination

To evaluate the performance of our model against RVE, three experienced independent experts, who did not participate in the labeling of Section “Calibration, patient and image selection”. Calibration, patient and image selection, reviewed 400 CAG of the total set. The mean and SD of the results of the evaluation metrics are presented in Table 3.

**Table 3:** Classification metrics for detecting coronary artery tortuosity in radiological visual estimation performed by 3 independent experts using 400 coronary angiographies.

Metric	Mean	Standard deviation
Accuracy	0.85	0.03
Sensitivity	0.84	0.02
Specificity	0.86	0.04
PPV	0.87	0.05
NPV	0.84	0.02
F_1_	0.85	0.03

### Saliency maps evaluation

**Figure 2:**
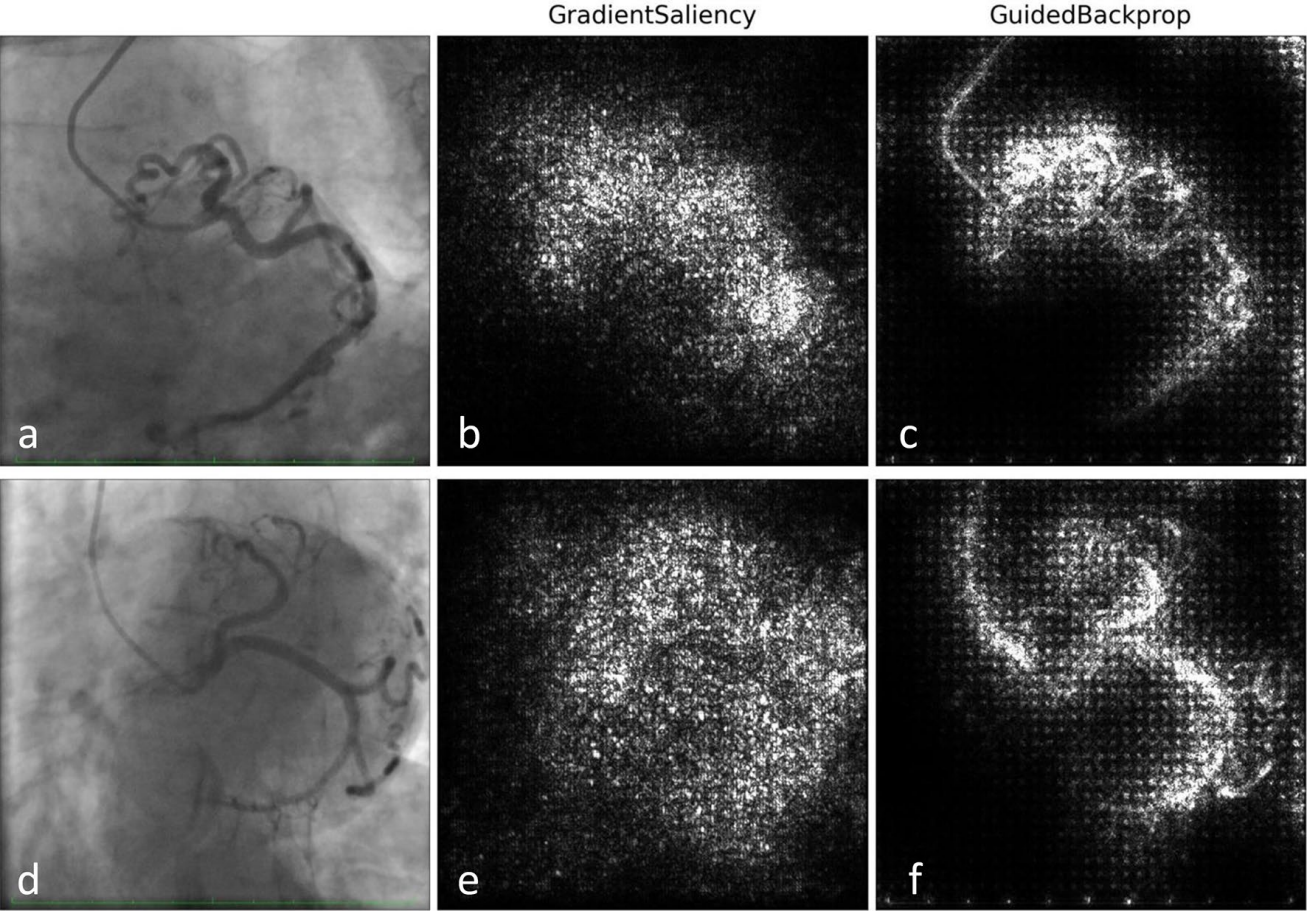
Saliency maps examples of left (Spider) coronary angiographies. (**a**)–(**c**) Patient with coronary artery tortuosity. Predicted labels: tortuous (99.8%), non-tortuous (0.2%). (**d**)–(**f**) Patient without coronary artery tortuosity. Predicted labels: non-tortuous (92.5%), tortuous (7.5%).

**Figure 3:**
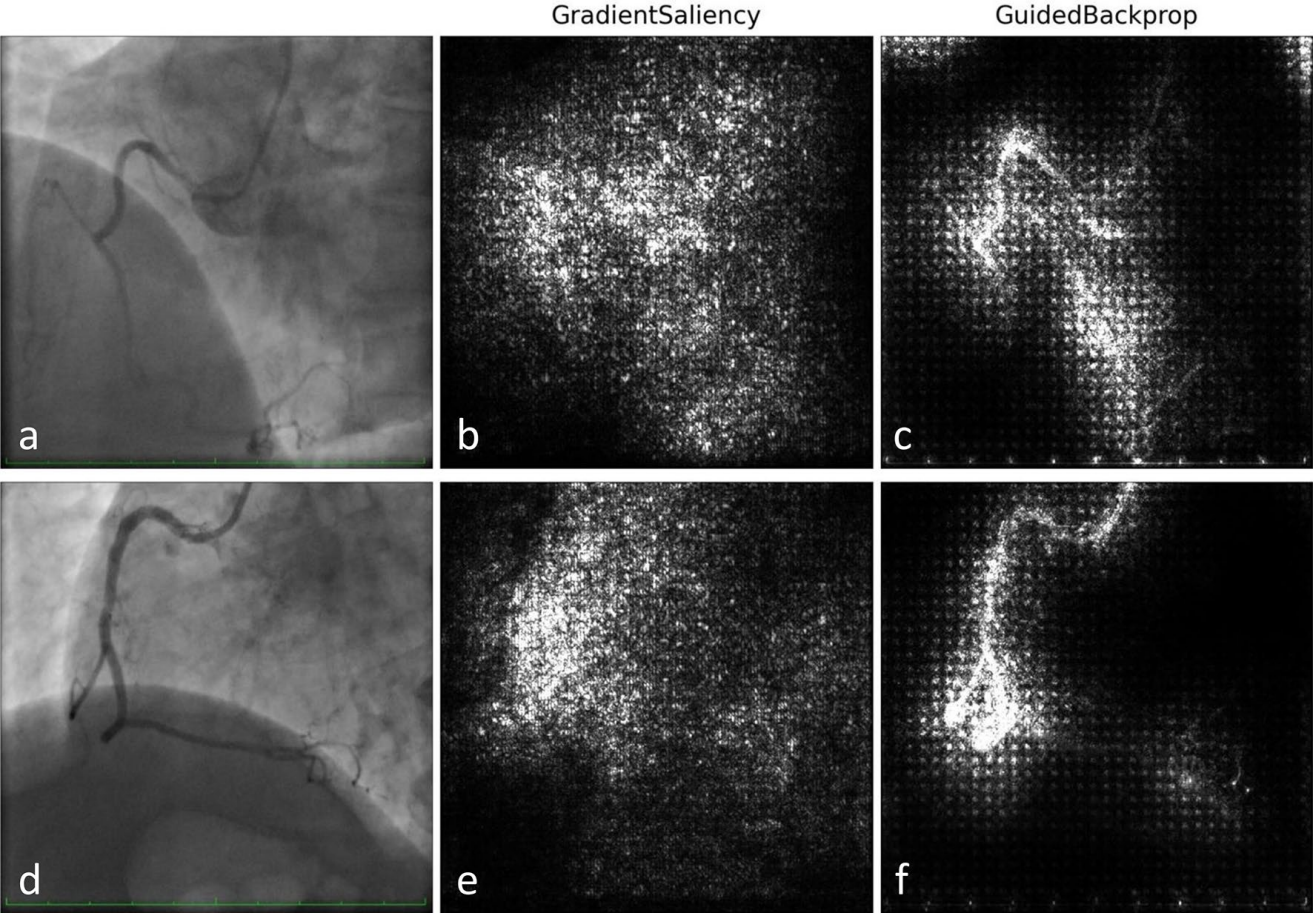
Saliency maps examples of right (45°*/*0°) coronary angiographies. (**a**)–(**c**) Patient with coronary artery tortuosity. Predicted labels: tortuous (83.9%), non-tortuous (16.1%). (**d**)–(**f**) Patient without coronary artery tortuosity. Predicted labels: non-tortuous (98.9%), tortuous (1.1%).

In this section we present saliency maps for four sample test images, with the aim of detecting those parts of the CAG image on which the model focused to perform the prediction. We represent gradient saliency (also known as vanilla gradient) and guided backpropagation^26,27^ in its standard version^28^, as illustrated in Figs. 2 and 3. The explanations provided by the saliency maps in both Figs. 2 and 3 highlight the artery region, especially in guided backpropagation. We have shown two examples of each side of CAG (Spider and 45°*/*0°) and the corresponding predictions.

## Discussion

The demand for medical imaging has progressively increased, but medical image analysis is challenging and time-consuming due to the shortage of radiologists^29^. Hence, new methods, such as DL systems, can be applied to automate multiple tasks in medical imaging^30^. The strengths of DL extend to improving clinical decision-making, identifying new phenotypes, and selecting better treatment strategies in complex diseases, including cardiovascular medicine^30^.

### Strengths of the proposed deep learning system

In this study, we report for the first time to our knowledge the use of DL techniques to detect CAT in CAG. The results demonstrate that our CNN-based models have sensitivity and specificity compatible with expert RVE for detecting CAT, with a sensitivity of (87 ± 10) % versus (84 ± 2) %, and a specificity of (88 ± 10)% versus (86 ± 4)%, respectively, for a conservative threshold of 0.5 in our DL system.

Furthermore, expert cardiologists observe the entire radiographic sequence for visual detection of CAT while our deep learning model only requires a single representative image of each angiographic projection at the point of maximum arterial filling with contrast material. The selection of the image with the highest contrast is a minor problem, which can be solved either with Artificial Intelligence methods, or with classical image analysis techniques, i.e. selecting the image with the highest number of pixels with Hounsfield units above a certain threshold.

The proposed DL model offers a seamless integration into clinical practice by leveraging the image sequences acquired and recorded during coronary angiography. With its ability to automatically detect tortuosity, this method provides valuable information that is currently not routinely obtained. This information proves essential in assessing vascular risks for patients with coronary artery tortuosity (CAT). By incorporating the DL model into regular practice, healthcare professionals can enhance their ability to identify and manage potential risks associated with CAT more effectively. Indeed, moderate/severe CAT is associated with higher rates of target vessel failure due to higher rates of target vessel-related myocardial infarction and ischemia-induced target vessel revascularization^31^. In addition, CAT is highly prevalent in spontaneous coronary artery dissection (SCAD) and is associated with recurrent SCAD^12^.

Our DL method enables automated CAT detection, which could have a beneficial impact on preventing cardiac lesions, shortening CAG examination times, establishing vascular risks in patients with CAT and improving future treatment strategies. Besides, the present study can be extended for future applications in industry by installing AI algorithms on devices currently used to recognize CAT in CAG images, which at the moment is a condition rarely reported by cardiologists^10^. Moreover, the DL methods applied in this study can be adapted and reproduced in other vascular beds.

### Limitations and weaknesses

The main limitations of the proposed method are reasonable image quality, pixel resolution and sufficient variety of CAT images containing several types of vascular tortuosity. In a subsequent study with a greater number of images, CAG images misclassified by the DL method could be evaluated for further validation of our system to assess whether there are consistent patterns in those CAG images that cause the model to misclassify them.

### Future work

Medical imaging plays a key role in medicine for monitoring, diagnosis, and treatment evaluation. Recent advances in DL have shown their potential utility in patient triage and assessment, particularly in medical imaging, where convolutional neural networks are suitable for several tasks, such as classification, segmentation, object detection or registration^32^. We have presented a novel DL system for detecting CAT in CAG images. Overall, our results demonstrate that the proposed DL model has comparable sensitivity and specificity in CAT detection with expert RVE for a conservative threshold of 0.5. By adapting the threshold of our DL algorithm, we believe that it can serve as a first screening to predict a patient’s likelihood of being diagnosed with CAT, providing additional assistance to specialist cardiologists in their work. Furthermore, we believe that this study could help to further validate future applications of AI techniques in cardiology.

## Supplementary Information

Supplementary Table A1.

## Data Availability

The data that support the findings of this study are not available for privacy reasons. Nevertheless, they can be available from the corresponding author upon reasonable request. The code is publicly available at https://github.com/MiriamCobo/CoronaryArteries.git.

## References

[1] GBD Eastern Mediterranean Region Cardiovascular Disease Collaborators (2018). Burden of cardiovascular diseases in the Eastern Mediterranean Region, 1990–2015: Findings from the Global Burden of Disease 2015 study. Int. J. Public Health.

[2] Benjamin EJ, Blaha MJ, Chiuve SE (2017). Heart disease and stroke statistics—2017 update: A report from the American Heart Association. Circulation.

[3] Wilkins, E., Wilson, L., Wickramasinghe, K. *et al.* European cardiovascular disease statistics 2017 (2017).

[4] Wielopolski P, van Geuns R, De Feyter P, Oudkerk M (2000). Coronary arteries. Eur. Radiol..

[5] Cuddy E, Robertson S, Cross S, Isles C (2005). Risks of coronary angiography. The Lancet.

[6] Sen T, Kilit C, Astarcioglu MA (2018). Comparison of quantitative and qualitative coronary angiography: Computer versus the eye. Cardiovasc. J. Afr..

[7] Ciurică S, Lopez-Sublet M, Loeys BL (2019). Arterial tortuosity: Novel implications for an old phenotype. Hypertension.

[8] Turgut O, Yilmaz A, Yalta K (2007). Tortuosity of coronary arteries: An indicator for impaired left ventricular relaxation?. Int. J. Cardiovasc. Imaging.

[9] Groves SS, Jain AC, Warden BE, Gharib W, Beto RJ (2009). Severe coronary tortuosity and the relationship to significant coronary artery disease. W. V. Med. J..

[10] Chiha J, Mitchell P, Gopinath B, Burlutsky G, Kovoor P, Thiagalingam A (2017). Gender differences in the prevalence of coronary artery tortuosity and its association with coronary artery disease. IJC Heart Vasc..

[11] Gaibazzi N, Rigo F, Reverberi C (2011). Severe coronary tortuosity or myocardial bridging in patients with chest pain, normal coronary arteries, and reversible myocardial perfusion defects. Am. J. Cardiol..

[12] Eleid MF, Guddeti RR, Tweet MS (2014). Coronary artery tortuosity in spontaneous coronary artery dissection: Angiographic characteristics and clinical implications. Circ. Cardiovasc. Interv..

[13] García ÁL, De Lucas JM, Antonacci M, Zu Castell W (2020). A cloud-based framework for machine learning workloads and applications. IEEE Access.

[14] Leung W-H, Stadius ML, Alderman EL (1991). Determinants of normal coronary artery dimensions in humans. Circulation.

[15] Caruana R, Lawrence S, Giles C (2000). Overfitting in neural nets: Backpropagation, conjugate gradient, and early stopping. Adv. Neural Inf. Process. Syst..

[16] Anguita, D., Ghelardoni, L., Ghio, A., Oneto, L. & Ridella, S. The ‘k’in k-fold cross validation. In *20th European Symposium on Artificial Neural Networks, Computational Intelligence and Machine Learning (ESANN)* 441–446, i6doc. com publ (2012).

[17] Chollet, F. Xception: Deep learning with depthwise separable convolutions (2017).

[18] Kingma, D. P. & Ba, J. Adam: A method for stochastic optimization (2017).

[19] Loshchilov, I. & Hutter, F. Decoupled weight decay regularization (2019).

[20] Zhuang F, Qi Z, Duan K (2021). A comprehensive survey on transfer learning. Proc. IEEE.

[21] Kim HE (2022). Transfer learning for medical image classification: A literature review. BMC Med. Imaging.

[22] Iglesias LL, Bellón PS, Del Barrio AP (2021). A primer on deep learning and convolutional neural networks for clinicians. Insights Imaging.

[23] Chollet, F. Keras. https://keras.io (2015).

[24] Abadi, M., Agarwal, A., Barham, P. *et al.* TensorFlow: Large-scale machine learning on heterogeneous systems. Software available from tensorflow.org (2015).

[25] Clark, A. Pillow (pil fork) documentation (2015).

[26] Simonyan, K., Vedaldi, A. & Zisserman, A. Deep inside convolutional networks: Visualising image classification models and saliency maps. arXiv preprint arXiv:1312.6034, (2013).

[27] Kim, B. *et al*. Why are saliency maps noisy? Cause of and solution to noisy saliency maps. In *2019 IEEE/CVF International Conference on Computer Vision Workshop (ICCVW)*. IEEE (2019).

[28] Anh, H. N. Deep-viz-keras GitHub Software by experiencor. https://github.com/experiencor/deep-viz-keras (2018).

[29] Puttagunta M, Ravi S (2021). Medical image analysis based on deep learning approach. Multimed. Tools Appl..

[30] Krittanawong C, Johnson KW, Rosenson RS, Wang Z (2019). Deep learning for cardiovascular medicine: A practical primer. Eur. Heart J..

[31] Konigstein M, Ben-Yehuda O, Redfors B (2021). Impact of coronary artery tortuosity on outcomes following stenting: A pooled analysis from 6 trials. JACC Cardiovasc. Interv..

[32] Greenspan H, Van Ginneken B, Summers RM (2016). Guest editorial deep learning in medical imaging: Overview and future promise of an exciting new technique. IEEE Trans. Med. Imaging.

