# Publisher Correction: Novel deep learning method for coronary artery tortuosity detection through coronary angiography

**DOI:** 10.1038/s41598-023-39135-0

**Published:** 2023-07-25

**Authors:** Miriam Cobo, Francisco Pérez-Rojas, Constanza Gutiérrez-Rodríguez, Ignacio Heredia, Patricio Maragaño-Lizama, Francisca Yung-Manriquez, Lara Lloret Iglesias, José A. Vega

**Affiliations:** 1 Advanced Computing and e-Science Research Group, Institute of Physics of Cantabria (IFCA), CSIC - UC, 39005 Santander, Cantabria, Spain; 2 Facultad de Medicina, Universidad Católica del Maule, Talca, Chile; 3 Departamento de Morfología y Biología Celular, Grupo de Investigación SINPOS, Universidad de Oviedo, 33006 Oviedo, Principality of Asturias Spain; 4 Facultad de Ciencias de la Salud, Universidad Autónoma de Chile, Talca, Chile; 5 Department of Hemodynamics, Talca Regional Hospital, Talca, Chile

Correction to: *Scientific Reports* 10.1038/s41598-023-37868-6, published online 10 July 2023

The original version of this Article contained errors in the Affiliations, where Francisco Pérez-Rojas was incorrectly affiliated with ‘Grupo de Investigación MEXPA, Facultad de Ciencias de la Salud, Universidad Autónoma de Chile, Talca, Chile.’ Their correct affiliations are listed below.

Facultad de Medicina, Universidad Católica del Maule, Talca, Chile.

Departamento de Morfología y Biología Celular, Grupo de Investigación SINPOS, Universidad de Oviedo, 33006, Oviedo, Principality of Asturias, Spain.

Additionally, Francisca Yung-Manriquez was incorrectly affiliated with ‘Facultad de Medicina, Universidad Católica del Maule, Talca, Chile.’ Their correct affiliation is listed below.

Facultad de Ciencias de la Salud, Universidad Autónoma de Chile, Talca, Chile.

The original Article has been corrected.

