# Supplementary material for: Novel deep learning method for coronary artery tortuosity detection through coronary angiography
Source: Sci Rep. 2023 Jul 10;13:11137. doi: 10.1038/s41598-023-37868-6 (PMC10333289; doi:10.1038/s41598-023-37868-6)
Supplement: Supplementary file 1 — Supplementary Table A1. [file 41598_2023_37868_MOESM1_ESM.docx]

**Supplementary Material**

**Appendix A.**

**Methods**

**Model design**

The five cross validation models were trained using early stopping and data augmentation in the training and validation sets. Data augmentation parameters are reported in Table A1.

**Table A1.** Detailed data augmentation parameters in training and validation.

| **Mode** | **Horizontal flip probability** | **Vertical flip probability** | **Rotation probability** | **Rotation limit** | **Random blur probability** | **Pixel saturation probability** |
| --- | --- | --- | --- | --- | --- | --- |
| Training | 0.5 | 0.5 | 0.5 | 90 | 0.1 | 0.1 |
| Validation | 0.5 | 0.5 | 0.5 | 90 | 0.1 | 0.1 |
